# Serum Neuroinflammatory Disease-Induced Central Nervous System Proteins Predict Clinical Onset of Experimental Autoimmune Encephalomyelitis

**DOI:** 10.3389/fimmu.2017.00812

**Published:** 2017-07-17

**Authors:** Itay Raphael, Johanna Webb, Francisco Gomez-Rivera, Carol A. Chase Huizar, Rishein Gupta, Bernard P. Arulanandam, Yufeng Wang, William E. Haskins, Thomas G. Forsthuber

**Affiliations:** ^1^Department of Biology, University of Texas at San Antonio, San Antonio, TX, United States; ^2^Division of Rheumatology and Clinical Immunology, Department of Medicine, University of Pittsburgh, Pittsburgh, PA, United States

**Keywords:** predictive biomarkers, multiple sclerosis, experimental autoimmune encephalomyelitis, T cell, central nervous system protein, serum expression

## Abstract

There is an urgent need in multiple sclerosis (MS) patients to develop biomarkers and laboratory tests to improve early diagnosis, predict clinical relapses, and optimize treatment responses. In healthy individuals, the transport of proteins across the blood–brain barrier (BBB) is tightly regulated, whereas, in MS, central nervous system (CNS) inflammation results in damage to neuronal tissues, disruption of BBB integrity, and potential release of neuroinflammatory disease-induced CNS proteins (NDICPs) into CSF and serum. Therefore, changes in serum NDICP abundance could serve as biomarkers of MS. Here, we sought to determine if changes in serum NDICPs are detectable prior to clinical onset of experimental autoimmune encephalomyelitis (EAE) and, therefore, enable prediction of disease onset. Importantly, we show in longitudinal serum specimens from individual mice with EAE that pre-onset expression waves of synapsin-2, glutamine synthetase, enolase-2, and synaptotagmin-1 enable the prediction of clinical disease with high sensitivity and specificity. Moreover, we observed differences in serum NDICPs between active and passive immunization in EAE, suggesting hitherto not appreciated differences for disease induction mechanisms. Our studies provide the first evidence for enabling the prediction of clinical disease using serum NDICPs. The results provide proof-of-concept for the development of high-confidence serum NDICP expression waves and protein biomarker candidates for MS.

## Introduction

Multiple sclerosis (MS) is an autoimmune demyelinating disease of the central nervous system (CNS), which affects over 2.5 million people globally and poses a substantial social and economic burden on the affected individuals and society (1, 2). MS is classified into four clinical subtypes, with relapsing–remitting MS (RRMS) initially being diagnosed in over 80% of patients (3). RRMS is characterized by periods of worsening of clinical symptoms (relapses) followed by partial or complete remissions (3). The etiology and pathogenic mechanisms of MS have been studied for over 100 years, and in the past decade, a number of novel therapies have been approved (2, 4). However, patients are still facing numerous clinical challenges including lack of early diagnosis, optimization of individual treatment plans, and paucity of laboratory tests to more accurately predict disease progression (2, 4). While early therapeutic intervention has shown some success in decreasing the frequency of disease relapses, its long-term impact and effect on conversion to secondary-progressive MS has yet to be established (5). Thus, there is an urgent need to develop biomarkers and laboratory tests to determine treatment efficacy, predict relapses, and provide measures of disease progression.

Traditionally, body fluids such as peripheral venous blood have been relatively easily accessible to measure biological markers in health and disease. For example, measurements of red blood cells, white blood cells, electrolytes, and lipids are commonly performed in clinical laboratory practice (6). Serum is a major component of blood and is composed of a plethora of proteins derived from diverse tissues (7). The vast array of serum proteins is specific to the blood; a majority of these proteins are produced by the liver and the gut, while less than one-third of the proteins found in serum are released by other tissues (7, 8). The serum levels of these so-called leakage-proteins are relatively stable in healthy individuals; significant changes in their levels are usually only observed as the result of tissue damage and disease conditions. For example, altered cardiac troponin levels are used as biomarkers to indicate the event of acute myocardial infarction (9).

Leakage of CNS proteins into the serum is tightly regulated by the blood–brain barrier (BBB) in healthy individuals (10). Importantly, during inflammatory events in MS, leukocytes such as autoimmune T cells are recruited to the CNS and produce cytokines and neurotoxic mediators, which ultimately induce tissue damage, increase the permeability of the BBB, and result in the potential release of neuroinflammatory disease-induced CNS proteins (NDICPs) into the blood, which could potentially provide biomarkers reflective of CNS pathologic events (11). The experimental autoimmune encephalomyelitis (EAE) model of MS has been extensively applied to the discovery of pathologic mechanisms of MS and for development and testing new therapies for MS (12). The disease course in EAE is very predictable, less heterogeneous, and significantly more synchronized as compared with the highly variable spectrum of disease in genetically diverse human MS patients. Therefore, the EAE model is a useful platform to develop and test novel biomarkers for CNS inflammatory and demyelinating diseases, and in particular MS (13).

Previously, we developed and applied microwave and magnetic (M2) proteomics to determine quantitative changes in the CNS proteome during EAE from brain tissue and pooled serum specimens from different phases of disease progression (14, 15). Importantly, we discovered protein expression waves, or expression trajectories, in CNS tissue that were characteristic for leading or lagging the clinical onset phase of EAE (15). Moreover, we showed that the identified CNS proteome changes classify EAE animals into risk groups determined by clinical measurement of disease severity (14, 15).

In the present study, we investigated a subset of NDICPs in serum sampled from individual mice during EAE for their predictive value for the onset of clinical disease. Importantly, we show that the crest (i.e., peak period) of the serum NDICP waves predicted the onset of clinical EAE signs (onset-leading wave) in individual mice and stratified healthy versus EAE subjects. The NDICPs identified in this study have human homologs and, therefore, may be translatable as biomarkers for investigations in human MS patients. These results provide proof-of-concept for the feasibility of developing predictive biomarkers for MS using altered serum levels of NDICPs. These putative biomarkers may improve early detection and intervention and accelerate drug development for MS.

## Materials and Methods

### Study Approval

All animals were maintained in specific pathogen-free conditions in the American Association for Laboratory Animal Science-accredited facility at the University of Texas at San Antonio. All experiments were approved by the Institutional Animal Care and Use Committee at the University of Texas at San Antonio and performed in accordance with the relevant guidelines and regulations. Mice were fed and watered *ad libitum*.

### Mice

C57BL/6 female mice were purchased from the Jackson Laboratory (Stock number 000664; Bar Harbor, ME, USA). 6–8 weeks old female mice were used in all experiments.

### EAE Induction

Active EAE was induced in C57BL/6 female mice by subcutaneous (s.c.) injection of 200 µg myelin oligodendrocyte glycoprotein (MOG)_35–55_ peptide (United Biochemical Research, Seattle, WA, USA) in 50 µL of complete Freund’s adjuvant (CFA) containing 5 mg/mL *Mycobacterium tuberculosis* H37Ra (Difco Laboratories, Detroit, MI, USA). Mice also received intraperitoneal injections of 400 ng *Bordetella pertussis* toxin (PTx) (List Biological, Campbell, CA, USA) on days 0 and 2. For induction of passive EAE by adoptive transfer, donor mice were immunized s.c. with 200 µg of MOG_35–55_ in CFA. Splenocytes and draining lymph node cells were collected from donor mice on day 10 and restimulated for 3 days at 37°C with 10 µg/mL of MOG_35–55_ peptide in complete DMEM containing 10% fetal bovine serum, 20 ng/mL of recombinant mouse IL-23 (R&D Systems, Minneapolis, MN, USA), and 10 µg/mL of anti-IFN-γ monoclonal antibody (mAb) (Bio X Cell, West Lebanon, NH; R4-6A2). Recipient mice were injected with 4,500 restimulated IL-17-producing donor cells.

### EAE Evaluation

Mice were assigned clinical scores for EAE according to the following scale (16): 0, no abnormality; 1, limp tail; 2, moderate hind limb weakness; 3, complete hind limb paralysis; 4, quadriplegia or premoribund state; 5, moribund, death.

### *Chlamydia muridarum* Infection

Mice were anesthetized intranasally using 3% isoflurane in a rodent anesthesia system (Harvard Apparatus, Holliston, MA, USA) and immediately inoculated intravaginally with 5 × 10^4^ inclusion-forming units (IFU) of *C. muridarum* in 5 µL of sterile SPG buffer, as previously described (17). Vaginal vaults of challenged mice were swabbed at 3-day intervals, and swabs were collected into Eppendorf tubes containing 4-mm glass beads (Kimble, Vineland, NJ, USA) and 500 µL of sterile SPG buffer. The tubes were vortexed for 1 min, and swab material was plated and incubated for 28 h with HeLa cells grown on coverslips in 24-well plates to facilitate *Chlamydia* replication and growth. The infected HeLa cells were fixed with 2% paraformaldehyde and permeabilized with 2% saponin. Cells were washed using PBS and incubated with DMEM containing 10% fetal bovine serum for 1 h to block non-specific binding. Thereafter, cells were washed and incubated with polyclonal rabbit anti-*Chlamydia* antibody for 1 h and then incubated for an additional 2 h with goat anti-rabbit immunoglobulin conjugated to fluorescein isothiocyanate (Sigma) plus Hoechst nuclear staining. The treated coverslip cultures were then washed and mounted onto Superfrost microscope slides (Fisher) by using FluorSave reagent (Calbiochem). Slides were visualized using a Zeiss Axioskop 2 Plus research microscope (Zeiss, Thornwood, NY, USA). The bacterial shedding was calculated and expressed as the number of IFU per animal.

### Cytokine ELISPOT Assay

Cytokine ELISPOT assay was performed and spots analyzed as described previously (18, 19). In brief, ELISPOT plates (Millipore, Billerica, MA, USA) were precoated with anti-mouse-IFN-γ mAb (eBioscience, AN-18) and anti-mouse-IL-17 mAb (Bio X Cell; 17F3). Splenocytes (5 × 10^5^ cells/well) and brain-infiltrating mononuclear cells (2–5 × 10^4^ cells/well) were restimulated with MOG_35–55_ peptide in HL-1 medium (Lonza) at 37°C for 24 h. Biotinylated anti-mouse-IFN-γ mAb (eBioscience; R4-6A2) and anti-mouse-IL-17 mAb (BioLegend, TC11-8H4) were then added overnight at 4°C, followed by incubation with streptavidin alkaline phosphatase (Invitrogen, Waltham, MA, USA) for 2 h at room temperature and developing with BCIP/NBT Phosphatase Substrate (KPL, Gaithersburg, MD, USA). After plate developing, image analysis of spots was performed on a Series 2 Immunospot analyzer (Cellular Technology Limited, Cleveland, OH, USA). Results for antigen-specific spot-forming cells were normalized with a negative control containing peptide-free media. All measurements were performed in duplicate or triplicate.

### Flow Cytometry and Antibodies

Single-cell suspensions were obtained from brain tissues by mechanical isolation. Extracellular myelin was removed from all brain and spinal cord suspensions using myelin removal beads (Miltenyi Biotec), according to the manufacturer’s instructions. For surface staining, cell suspensions were blocked with 1% anti-mouse CD16/CD32 (eBioscience; 93) for 20 min on ice and then stained with fluorescently labeled antibodies for murine CD4 (BD Biosciences; RM4-5), CD11c (eBioscience; N418), CD11b (eBioscience; M1/70), or GR1 (BD Biosciences; RB6-8C5), for 45 min. Following staining, cells were washed with PBS, lysed for red blood cell (Beckman Coulter), and fixed using a fixative agent (eBioscience). All samples were analyzed or sorted on a BD FACSAria II (BD Bioscience). Data were analyzed using the FlowJo 10 Data Analysis software (FlowJo LLC) and BD FACSDiva software (BD Biosciences).

### Brain Tissue Lysate

Protein was extracted from whole mouse brain using the RIPA Lysis Buffer Kit (Santa Cruz Biotechnology) as per the manufacturer’s protocol. Briefly, an appropriate amount of RIPA complete lysis buffer was added to cell pellet. The mixture was incubated on ice for 5 min, followed by centrifugation at 14,000 × *g* for 15 min at 4°C. The supernatant was collected as brain tissue lysate and stored at −80°C until further use. Protein concentration was determined using Invitrogen EZQ Protein Quantitation Kit (Invitrogen) and Pierce BCA Protein Assays (ThermoFisher Scientific). Protein from all mice (*n* = 157), spanning all time points, was pooled as reference material.

### Serum Collection

Samples were obtained from a single cheek-puncture for each individual mouse. Whole blood samples were collected using BD Vacutainer SST Blood Collection Tubes (BD Worldwide; Product Number: 367381) for serum separation as described previously (20). Specimens were centrifuged at 1,100 × *g* for 20 min after clotting for 30 min.

### Enzyme-Linked Immunosorbent Assay (ELISA)

Commercial ELISA Kits for synapsin-1 (ABIN426058), synapsin-2 (ABIN852365), synaptotagmin-1 (ABIN1117297), neuronal enolase (ABIN1116159), glutamine synthetase (ABIN1570168), protein deglycase DJ-1 (ABIN819944), and NEFL (ABIN427109) were used as per the manufacturer’s suggested protocol (Antibodies-online.com Inc.). For brain tissue specimens, 300–500 µg of protein per sample was added to the corresponding well in each plate. For serum specimens, 40 µL of serum and 60 µL of sample-diluent per sample were added to the corresponding well in each plate. Plates were read at OD 450 nm for absorbance on a Synergy HT microplate reader (BioTek). Data were analyzed using the BioTek Gen5 software.

### Blinded Sample Group Allocation

Experimental autoimmune encephalomyelitis was induced, serum was collected from naive and EAE mice, and a randomly generated number assigned to the samples. The serum concentration of protein biomarker candidates was then determined by ELISA by a second investigator blinded to the origin of the samples. The serum concentration of protein biomarker candidates was then used to stratify/predict EAE versus naive animals by the second and/or third investigator.

### M2 Sample Preparation

Microwave and magnetic proteomics were performed as previously described (14, 15). For isobaric tandem mass tagging (TMT) labeling, protein was pooled from all specimens by protein amount as reference material prior to sample preparation from individual specimens. A total of 50 mg of C8 magnetic beads (BcMg; Bioclone Inc.) were suspended in 1 mL of 50% methanol. Immediately before use, 100 µL of the beads were washed three times with equilibration buffer [200 mM NaCl, 0.1% trifluoroacetic acid (TFA)]. Whole cell protein lysate (25–100 µg at 1 µg/µL) was mixed with pre-equilibrated beads and one-third sample binding buffer (800 mM NaCl, 0.4% TFA) by volume. The mixture was incubated at room temperature for 5 min followed by removing the supernatant. The beads were washed twice with 150 µL of 40 mM triethylammonium bicarbonate (TEAB), and then, 150 µL of 10 mM dithiothreitol (DTT) was added. The bead–lysate mixture underwent microwave heating for 10 s. DTT was removed and 150 µL of 50 mM iodoacetamide added, followed by a second microwave heating for 10 s. The beads were washed twice and re-suspended in 150 µL of 40 mM TEAB. *In vitro* proteolysis was performed with 4 µL of trypsin in a 1:25 trypsin-to-protein ratio (stock = 1 µg/µL in 50 mM acetic acid) and microwave heated for 20 s in triplicate. The supernatant was used immediately or stored at −80°C. Released tryptic peptides from digested protein lysates, including the reference materials described above, were modified at the N-terminus and at lysine residues with the TMT-6plex isobaric labeling reagents (ThermoFisher Scientific). Each individual specimen was encoded with one of the TMT-127-130 reagents, while reference material was encoded with the TMT-126 and -131 reagents: 41 µL of anhydrous acetonitrile was added to 0.8 mg of TMT labeling reagent for 25 µg of protein lysate and microwave heated for 10 s. To quench the reaction, 8 µL of 5% hydroxylamine was added to the sample at room temperature. To normalize across all specimens, TMT-encoded cell lysates from individual specimens, labeled with the TMT-127-130 reagents, were mixed with the reference material encoded with the TMT-126 and -131 reagents in 1_126_:1_127_:1_128_:1_129_:1_130_:1_131_ ratios. These sample mixtures, including all TMT-encoded specimens, were stored at −80°C until further use.

### Capillary Liquid Chromatography-Fourier-Transform-Tandem Mass Spectrometry (LC/FT/MS/MS) with Protein Database Searching

Capillary LC/FT/MS/MS was performed with a split less nanoLC-2D pump (Eksigent, Livermore, CA, USA), a 50 μm-i.d. column packed with 7 cm of 3 µm-o.d. C18 particles, and a hybrid linear ion trap-Fourier-transform tandem mass spectrometer (LTQ-ELITE; ThermoFisher, San Jose, CA, USA) operated with a lock mass for calibration. The reverse-phase gradient was 2–62% of 0.1% formic acid in acetonitrile over 60 min at 350 nL/min. For unbiased analysis, the top six most abundant eluting ions were fragmented by data-dependent HCD with a mass resolution of 120,000 for MS and 15,000 for MS/MS. For isobaric TMT labeling, probability-based protein database searching of MS/MS spectra against the TrEMBL protein database (release December 29, 2012; 59,862 sequences) was performed with a 10-node MASCOT cluster (v. 2.3.02; Matrix Science, London, UK) with the following search criteria: peak picking with Mascot Distiller; 10 ppm precursor ion mass tolerance, 0.8 Da product ion mass tolerance, three missed cleavages, trypsin, carbamidomethyl cysteines as a static modification, oxidized methionine and deamidated asparagine as variable modifications, an ion score threshold of 20, and TMT-6-plex for quantification.

### Pathway and Network Analysis

Function, network, and pathway analysis were performed with QIAGEN ingenuity pathways analysis (IPA) according to the manufacturer’s suggestions. Briefly, quantile MASCOT results were imported to IPA and IPA’s core analysis was performed on each data set. Differentially expressed proteins from the preclinical onset time points were mapped onto diseases and functions, canonical signaling pathways, and molecular networks. Quantified proteins in each category were visualized to investigate function, pathway and molecular network enrichment during these time points, where *P* values for dysregulation and enrichment were assigned by IPA.

### Statistics and Software

Statistical analysis for relative expression change of proteins revealed by M2 proteomics was performed as described previously (15). M2 proteomics results for each technical replicate estimate peptide expression for individual mice, encoded in sample mixtures, relative to pooled reference material from all mice, and spanning all time points. Relative peptide expression levels were transformed to log base 2 for quantile normalization. We tested for changes in relative peptide expression with post-immunization time using a linear mixed-effect model in which time was treated as a multilevel factor. We tested all the pairwise differences in relative peptide expression between all disease time points, using an unpaired, unequal variance *t*-test on the replicate averages. Statistical analysis was performed with R v3.0.2 (R-Project, Vienna, Austria).

Additional statistical analysis, including Student’s *t*-test, ANOVA, and receiver operating characteristic (ROC), were performed using GraphPad Prism 7 (GraphPad Software) and SigmaPlot 12 (Systat Software Inc.) software. Statistical significance was determined as indicated in the text and/or figure legends. Unless otherwise indicated, normal distribution was assumed for all samples. *P* values: *≤0.05; **≤0.01; ***≤0.001; ****≤0.0001.

## Results

### A Characteristic CNS Proteome Expression Wave Precedes Clinical Onset of EAE

**Figure 1:**
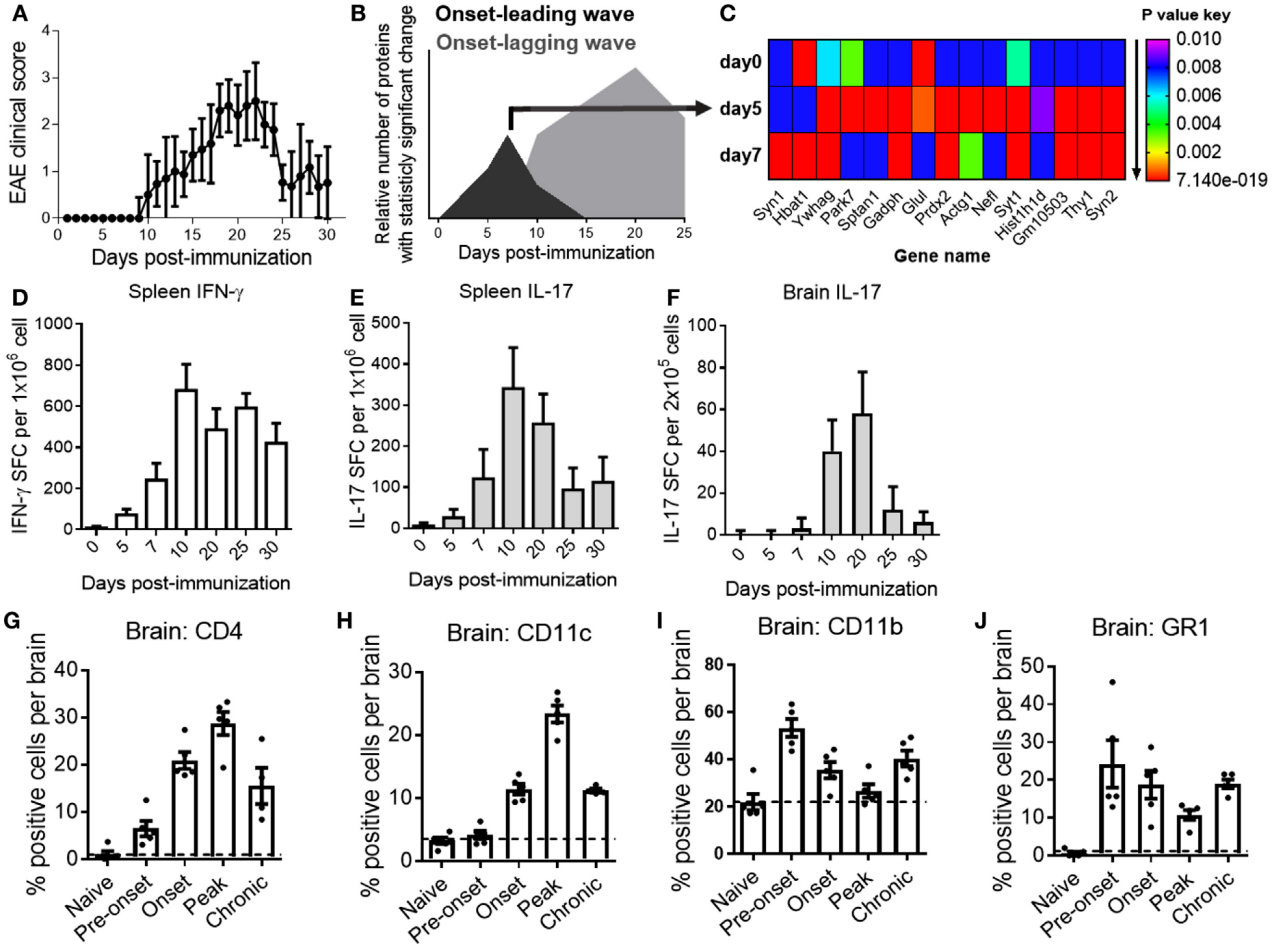
A characteristic central nervous system (CNS)-proteome expression wave precedes onset of clinical experimental autoimmune encephalomyelitis (EAE) and infiltration of inflammatory cells into the CNS. **(A)** EAE clinical disease scores of C57BL/6 mice. Data are shown as the mean clinical scores ± SD; *n* = 30. **(B)** Pre-onset and acute disease phase CNS proteome expression waves in EAE mice. Data are shown as the relative number of proteins with statistically significant fold change (*P* < 0.01) as compared with the total number of proteins in the dataset at the indicated time point after induction of disease. Black curve represents the onset-leading (pre-onset) wave (days 0–7). Gray curve represents the onset-lagging (clinical signs) wave (days 10–25). **(C)** Heat map of expression changes of 15 representative proteins with significant fold change (*P* = 0.01 to 7.14E−19) at the pre-onset phase. Shown are *P* values of the change in relative protein abundance versus reference material for naive mice (day 0) and pre-onset phase mice (days 5 and 7). Red is lowest; *P* value ≤ 1.15E−15, purple is highest; *P* value ≤ 0.01 **(D,E,F)** frequencies of myelin oligodendrocyte glycoprotein_35–55_-reactive T cells in spleen **(D,E)** and brain **(F)** of EAE mice at different time points. Data show the mean ± SD number of spot-forming cells measured by ELISPOT assay for IFN-γ **(D)** and IL-17 **(E,F)**. Shown are representative data from three independent experiments; *n* = 15. **(G–J)** Quantification of brain mononuclear cells by flow cytometry for CD4 **(G)**, CD11c **(H)**, CD11b **(I)**, and GR1 **(J)** at different phases of EAE. Data are shown as scatter-plot for individual measurements and the mean ± SEM. Shown are representative results of two to three independent experiments; *n* = 5–8. *Y*-axis grid line marks the basal-level (naive state) expression for each measured marker.

Clinical signs of MOG_35–55_ peptide-induced EAE in C57BL/6 mice, a standard EAE model, usually become apparent by 10–11 days post-immunization and disease peaks around days 19–22 (Figure 1A). Confirming previous studies, statistical analysis of the CNS proteome over the course of EAE by M2 proteomics revealed two subsets of protein expression waves with a statistically significant expression change specific for pre-onset (onset-leading wave; black curve) and acute disease phase (onset-lagging wave; gray curve) of EAE (Figure 1B) (14, 15). Statistical analysis revealed proteins that were significantly up- or downregulated within each time point at the onset-leading wave (Figure 1C).

To correlate the CNS protein expression waves with cellular and inflammatory events over the course of EAE, we determined T-cell cytokine production and inflammatory infiltrates at representative time points, including during the pre-onset phase of EAE (onset-leading phase) comprised induction of disease (day 0), pre-onset (days 5 and 7), and acute disease (onset-lagging phase) comprised onset (day 10), peak disease (day 20), and at remission (days 25 and 30).

In line with previously published results (15, 19), large frequencies of MOG-reactive IFN-γ and IL-17 producing Th1 and Th17 cells could be detected in secondary lymphatic tissues as early as day 5 after immunization and the numbers peaked after the onset of clinical disease (Figures 1D,E). Frequencies of pathogenic Th17 cells in the CNS mirrored the disease trajectory (Figure 1F). Analysis of CD4^+^ T cells, CD11c^+^ dendritic cells, and CD11b^+^ macrophages/microglia in the CNS by flow cytometry corresponded to inflammatory cytokine production and clinical EAE (Figures 1G–I), consistent with previous studies (21). Of note, a substantial number of CD11b^+^ (macrophages/microglia) cells and GR1^+^ cells (monocytes/neutrophils) were present at the pre-onset phase of EAE (Figures 1I,J).

Based on these results, we hypothesized that the EAE onset-leading CNS proteome expression wave may be a result of early (subclinical) CNS inflammation and could reveal candidate serum NDICP biomarkers to predict disease onset in EAE.

### Prioritization of Candidate Serum NDICP Biomarkers by Statistical and Bioinformatic Analysis

The CNS proteome expression profile during the pre-onset phase of EAE included nearly 800 proteins and over 2,000 unique peptides with altered expression, in agreement with our previous studies (14, 15). High-confidence serum NDICP expression waves and protein biomarker candidates for the prediction of disease onset were selected based on the following prioritization criteria: (i) Statistically significant difference (*P* ≤ 0.05) in expression compared to control during the preclinical (pre-onset) stage at either day 5 or day 7 post-immunization (*P* = 0.05 to 1.10E−43); proteins which did not achieve statistical significance during either one of these time points (day 5 or day 7) were excluded. (ii) Biological/functional relevance for neuroinflammatory and demyelinating diseases based on bioinformatic analysis of the pre-onset M2 proteomics datasets (Figure S1 in Supplementary Material), in accordance to previous studies (22). (iii) CNS tissue-specific or primary expression (i.e., NDICPs) based on bioinformatic analysis with tools provided by the EMBL-EBI expression atlas (23), MOPED (24), and ProteomicsDB (25). Ubiquitously expressed or non-primarily CNS-expressed proteins were excluded (e.g., actin, tubulin, and enzymes of glycolysis). (iv) A human homolog to increase the utility of the candidate biomarkers for studies in human MS patients.

**Figure 2:**
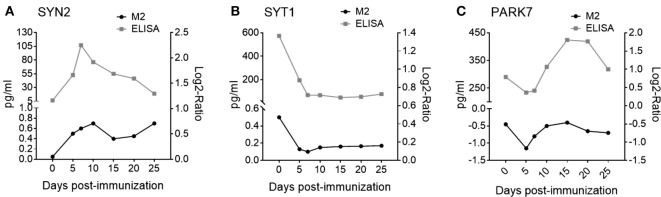
Microwave and magnetic (M2) proteomics protein expression trajectories show similar trends to protein concentrations in the central nervous system (CNS) during experimental autoimmune encephalomyelitis (EAE). Relative protein abundance in CNS tissue (black curves, bottom) measured by tandem mass spectrometry during EAE for **(A)** synapsin-2 (SYN2); Q8CE19_MOUSE peptide: SFRPDFVLIR, **(B)** synaptotagmin-1 (SYT1); Q3TPT3_MOUSE peptide: YVPTAGK, and **(C)** Protein deglycase DJ-1 (PARK7); B2KFH8_MOUSE peptide: ALVILAK. Data are shown as mean expression. Shown are results of one independent experiment; *n* = 160. Protein abundance in CNS tissue measured by enzyme-linked immunosorbent assay (ELISA) (gray curves, top) during EAE for **(A)** SYN2, **(B)** SYT1, and **(C)** PARK7. Data are shown as mean expression. Shown are results of two to three independent experiments; *n* = 5–15 per time point.

Shown in Table 1 are seven pre-onset phase NDICP biomarker candidates for predicting clinical onset after applying the selection criteria. These NDICPs included synapsin-2 (SYN2), synaptotagmin-1 (SYT1), neurofilament light (NEFL), glutamine synthetase (GS), protein deglycase DJ-1 (PARK7), enolase-2 (ENO2), and synapsin-1 (SYN1), which was previously reported by us (15). Additionally, we employed orthogonal ELISA-based measurements to confirm the expression waves for several NDICPs from the EAE CNS proteome that were revealed with M2 proteomics. Shown in Figure 2, the M2 proteomics results (black line, bottom) had similar expression trajectory trends to quantitative protein measurements by ELISA (gray line, top) for SYN2, SYT1, and PARK7. Statistical analysis showed a strong correlation of ELISA and M2 proteomics measurements for SYT1 and PARK7 and, to a lesser extent, for SYN2 (Figure S2 in Supplementary Material). Other proteins, which also showed similar expression trends during the pre-onset phase of EAE, showed a partial correlation trend between relative abundance (M2 proteomics) and concentration (ELISA) in CNS during the acute phase of the disease (days 10–25) (Figure S3 in Supplementary Material).

**Table 1:** Putative serum neuroinflammatory disease-induced central nervous system proteins biomarker candidates revealed with microwave and magnetic proteomics analyses.

Protein symbol (UniProt accession)	Protein common name(s)	Most statistically significant peptide sequence (based on overall *P* value)	Overall *P* value	Fold change and *P* value at day 5	Fold change and *P* value at day 7	Relevant GO annotations (function; process; cellular component)
SYN2 (Q8CE19_MOUSE)	Synapsin-2	SFRPDFVLIR	1.30E−18	16.33	21.33	Cytoskeletal protein binding; regulation of neurotransmitter secretion; myelin sheath and synaptic vesicle
				*P* = 1.80E−05	*P* = 1.00E−07	
SYN1 (A2AE14_MOUSE^a^)	Synapsin-1	MGHAHSGMGK	9.00E−18	0.14	1.83	Catalytic activity; neurotransmitter secretion; axon, synaptic vesicle, and myelin sheath
				*P* = 3.40E−02	*P* = 1.20E−12	
SYT1 (Q3TPT3_MOUSE)	Synaptotagmin-1	VFVGYNSTGAELR	2.50E−14	−1.35	−1.28	Lipoprotein receptor binding; glutamate secretion and synaptic transmission; excitatory synapse
				*P* = 4.40E−06	*P* = 1.70E−05	
NEFL (Q05DD2_MOUSE)	Neurofilament, light polypeptide; NF-L	KGADEAALAR	2.08E−12	4.39	1.36	Structural constituent of cytoskeleton; regulation of neuron apoptotic process, neurofilament cytoskeleton organization, locomotion; intermediate filament and myelin sheath
				*P* = 7.87E−09	*P* = 7.52E−02	
GS\GLUL (D3YVK1_MOUSE)	Glutamine synthetase; glutamate-ammonia ligase	LVLCEVFK	9.90E−05	9.42	8.40	Glutamate binding and ligase/lyase activity; glutamate metabolic process, axon terminus, myelin sheath and neuron projection
				*P* = 1.00E−02	*P* = 9.00E−02	
PARK7 (B2KFH8_MOUSE^b^)	Protein deglycase DJ-1; Parkinson protein 7	ALVILAK	6.40E−03	−1.80	−1.05	Peroxide acceptor oxidoreductase, transcription co-regulator; response to oxidative stress, negative regulation of neuron apoptotic process; extracellular vesicular exosome and axon
				*P* = 2.00E−02	*P* = 2.10E−02	
ENO2 (D3Z2S4_MOUSE)	Enolase-2; gamma-enolase; neural enolase	GNPTVEVDLYTAK	6.90E−03	−7.23	−4.29	Lyase activity; glucose metabolic process and response to drug; myelin sheath and neuronal cell body
				*P* = 3.00E−03	*P* = 1.00E−03	

*Shown are seven proteins, which were selected as predictive biomarker candidates for EAE to be further analyzed in serum. Proteins are sorted based on “overall *P* value” (top is lowest). Shown is fold change for the indicated time point versus day 0 (naive mice)*.

*^a^Entry name changed to O88935_MOUSE*.

*^b^Entry name changed to Q99LX0_MOUSE*.

In summary, these results, together with our previously published data (14, 15), suggest that NDICP expression analysis with M2 proteomics is an efficient means to discover tissue-specific expression changes during the pre-onset phase of the disease. Moreover, these results provide evidence for the utility of applying a statistical and bioinformatic prioritization strategy to M2 proteomics results to identify novel protein biomarker candidates in EAE.

### The Pre-Onset Phase of EAE Shows a Characteristic Serum NDICP Wave

To determine the utility of the NDICP biomarker candidates revealed by our analysis (Table 1) for predicting onset of clinical disease, we induced EAE and collected serum longitudinally from these animals and from matched naive control mice at multiple time points corresponding to different clinical stages of disease. We then analyzed these samples for our high-confidence NDICP serum expression waves and protein biomarker candidates with ELISA.

**Figure 3:**
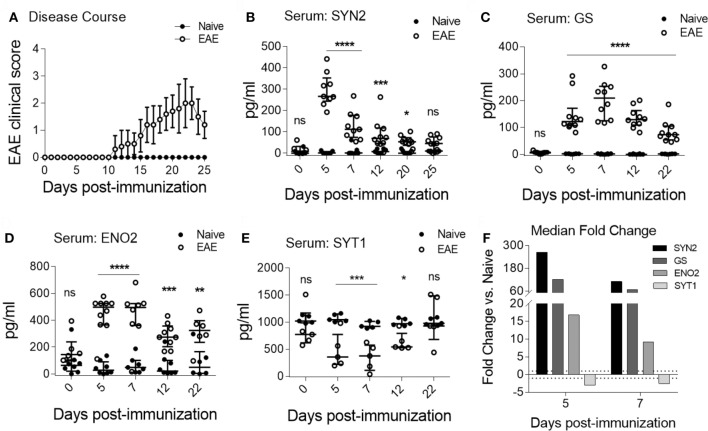
Characteristic serum neuroinflammatory disease-induced central nervous system protein wave at pre-onset of experimental autoimmune encephalomyelitis (EAE). **(A)** Shown are EAE clinical disease scores of C57BL/6 in mice immunized for EAE (open symbols) and naive controls (closed symbols). Data are shown as the mean clinical scores ± SD; *n* = 15 per group. **(B–E)** Serum protein abundance measured by enzyme-linked immunosorbent assay in EAE (open symbols) and naive mice (closed symbols) for synapsin-2 (SYN2) **(B)**, glutamine synthetase (GS) **(C)**, enolase-2 (ENO2) **(D)**, and synaptotagmin-1 (SYT1) **(E)**. Data show median ± IQR with individual measurements. Shown are results of three to four independent experiments with *n* = 8–15 per group for EAE; *n* = 5 for naive. **(F)** Shown is the median serum fold change for SYN2, GS, ENO2, and SYT1 at days 5 and 7 (Student’s *t*-test, *P* value: *≤0.05; **≤0.01; ***≤0.001; ****≤0.0001; ns, not significant).

As shown in Figure 3A, clinical EAE became apparent by 11 days post-immunization. Importantly, we observed specific serum NDICP expression patterns in EAE mice for SYN2, GS, ENO2, and SYT1 (Figures 3B–E). Specifically, the serum levels for SYN2, GS, and ENO2 were increased at the pre-onset phase of the disease, whereas the levels for SYT1 were decreased (Figures 3B–E). Notably, while the expression of these NDICPs was most strikingly altered during the pre-onset phase of EAE, they showed a trend toward returning to baseline levels at onset and peak disease (Figures 3B–E). Serum levels of these NDICPs showed statistically significant changes from the levels of naive mice for the same time points, particularly at the pre-onset phase of EAE (Figures 3B–E). Of note, serum levels of SYN2 peaked at day 5 after induction of EAE, whereas serum levels of GS peaked at day 7 (Figures 3B,C). Serum levels of ENO2 and SYT1 at days 5 and 7 post-immunization were similar (Figures 3D,E). Shown in Figure 3F is the serum fold change for the respective proteins at each pre-onset time point from EAE mice versus baseline levels of naive mice. Figure 3F shows that the magnitude of change in serum levels was strongly affected by EAE, particularly for SYN2 and GS, which showed over 50-fold change increase. Notably, the changes in serum protein levels for SYN2, ENO2, and SYT1 during EAE correlated with the expressions changes in the CNS (Figures 2 and 3, and not shown for ENO2). In contrast, the serum levels of other NDICP biomarker candidates (Table 1), including SYN1, NEFL, and PARK7, were not significantly altered during disease (Figure S4A–C in Supplementary Material; respectively). Importantly, in naive mice, serum levels of these NDICPs remained stable over time and no significant expression changes were noted within the same individuals during the observation period (Figures 3B–E, and not shown for SYN1, NEFL, and PARK7). Taken together, our results suggest that the preclinical onset (or onset-leading) phase of EAE is characterized by specific serum NDICP expression waves as indicated by altered serum abundance of SYN2, GS, ENO2, and SYT1.

### Blinded Confirmation of EAE Onset-Leading Serum NDICP Expression

**Figure 4:**
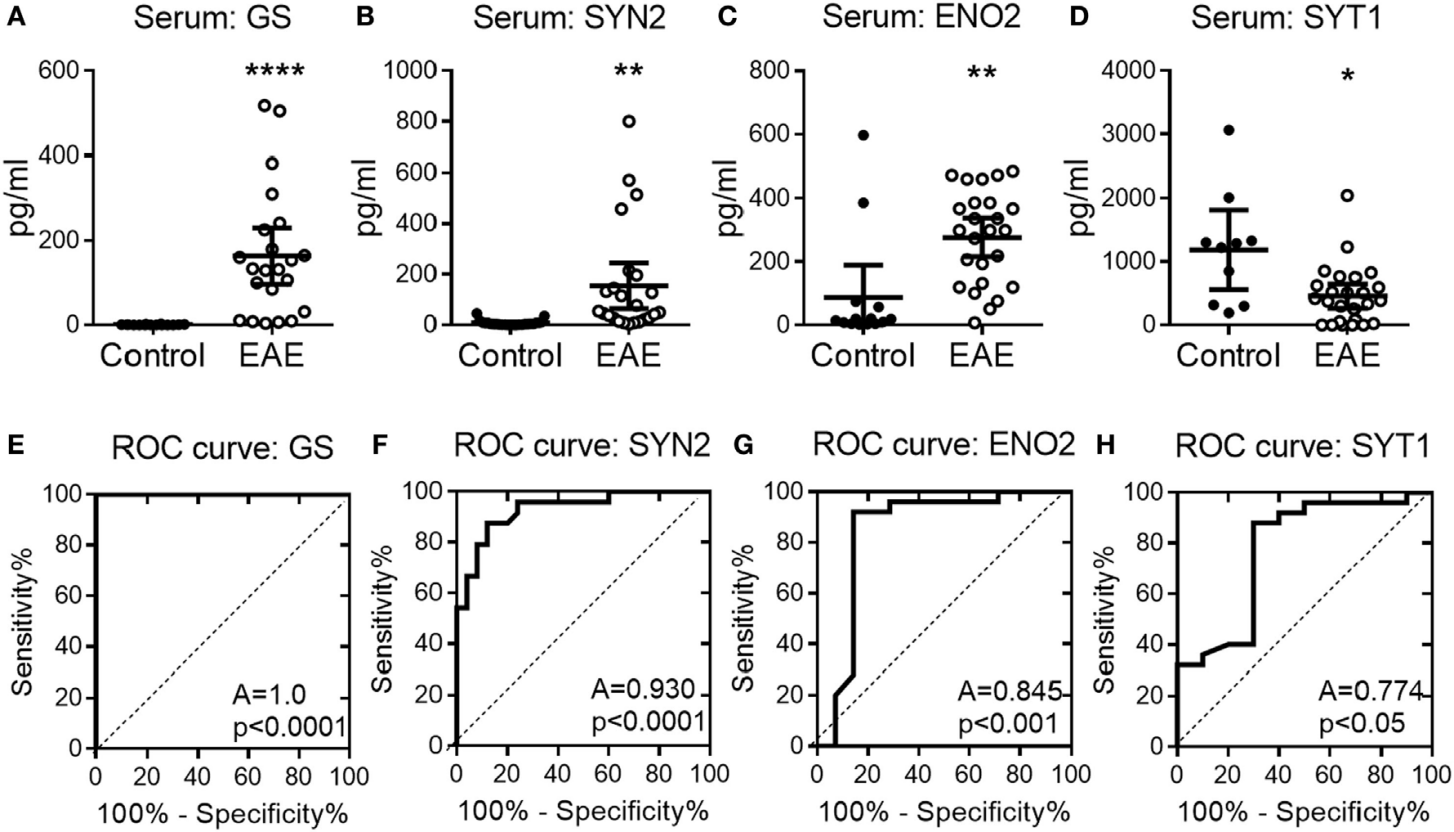
Serum abundance of experimental autoimmune encephalomyelitis (EAE) onset-leading neuroinflammatory disease-induced central nervous system proteins in individual mice. **(A–D)** Shown is serum protein abundance measured by enzyme-linked immunosorbent assay at the pre-onset phase of EAE (open symbols) and naive mice (closed symbols) for glutamine synthetase (GS) **(A)**, synapsin-2 (SYN2) **(B)**, enolase-2 (ENO2) **(C)**, and synaptotagmin-1 (SYT1) **(D)**. Data are shown as mean with 95% CI of scatter-plot for individual measurements. **(E–H)** Receiver operating characteristic (ROC) analysis curves for the serum protein concentration of GS **(E)**, SYN2 **(F)**, ENO2 **(G)**, and SYT1 **(H)**. *A*, area under the ROC curve. Shown are results pooled for *n* ≥ 8 animals per group over three independent experiments (unpaired two-tailed *t*-test with Welch’s correction, *P* value: *≤0.05; **≤0.01; ****≤0.0001).

To confirm the potential of serum NDICP expression waves and protein biomarker candidates as predictive tools for EAE onset, serum was collected at various time points from EAE mice and analyzed for the abundance of GS, SYN2, ENO2, and SYT1 by an investigator blinded to the EAE results. Shown in Figure S5 in Supplementary Material are EAE scores for three independent experiments and naive control animals, where serum was collected during the pre-onset phase of disease from EAE and naive animals (indicated by arrows). Shown in Figures 4A–D are serum levels of GS, SYN2, ENO2, and SYT1, respectively, in individual mice immunized for EAE (right panels; open circles) as compared with naive animals (left panels; filled circles). Importantly, serum levels of GS, SYN2, ENO2, and SYT1 stratified with 100%, 78%, 85%, and 80% accuracy, respectively, for healthy versus EAE animals and predicted the development of clinical EAE. Additionally, serum levels of GS and SYN2 were tightly controlled in naive mice (Figures 4A,B), whereas the levels of ENO2 and SYT1 showed more biological spread across multiple experiments (Figures 4C,D).

Next, we determined the sensitivity and specificity of the serum NDICP biomarker candidates and their predictive value by calculating ROC values. This model has been widely used to assess the sensitivity/specificity and predictive values of novel biomarkers (26). Using ROC analysis, we determined the discriminating power of GS, SYN2, ENO2, and SYT1 during the pre-onset phase of EAE as predictors for disease development. The results suggested that GS had the highest probability to discriminate between animals with EAE and healthy controls when analyzing serum samples from animals with unknown disease induction status (100%; i.e., no risk), followed by SYN2 (93%), ENO2 (85%), and SYT1 (77%), respectively (Figures 4E–H; percentages reflect AUC).

Taken together, the results strongly support the notion that the disease onset-leading serum expression wave of GS, SYN2, ENO2, and SYT1 can predict EAE clinical onset and discriminate between EAE mice versus healthy control animals.

### The Effect of Adjuvants and Infection on the Serum Abundance of EAE Onset-Leading NDICPs

Induction of active disease in EAE models requires the injection of neuroantigens in CFA and *Bordetella* PTx (27). Previously, it was reported that injection of CFA and PTx in mice triggers a complex set of signals in innate immune cells (16, 28). Moreover, adjuvant-induced signals can result in an increased BBB permeability and potentially promote the leakage of NDICPs into the blood circulation (29–33). Therefore, we investigated the effect of adjuvants and infection on the abundance of EAE onset-leading NDICPs in serum. Serum was collected during the pre-onset phase of disease (days 5 and 7 post-immunization) from individual naive mice, or animals immunized for EAE with MOG_35–55_:CFA, CFA alone, and PTx alone. Additionally, serum was collected from individual mice in a *C. muridarum* infection model to investigate whether an infectious disease model that does not directly affect the CNS could alter the serum levels of EAE onset-leading NDICPs.

**Figure 5:**
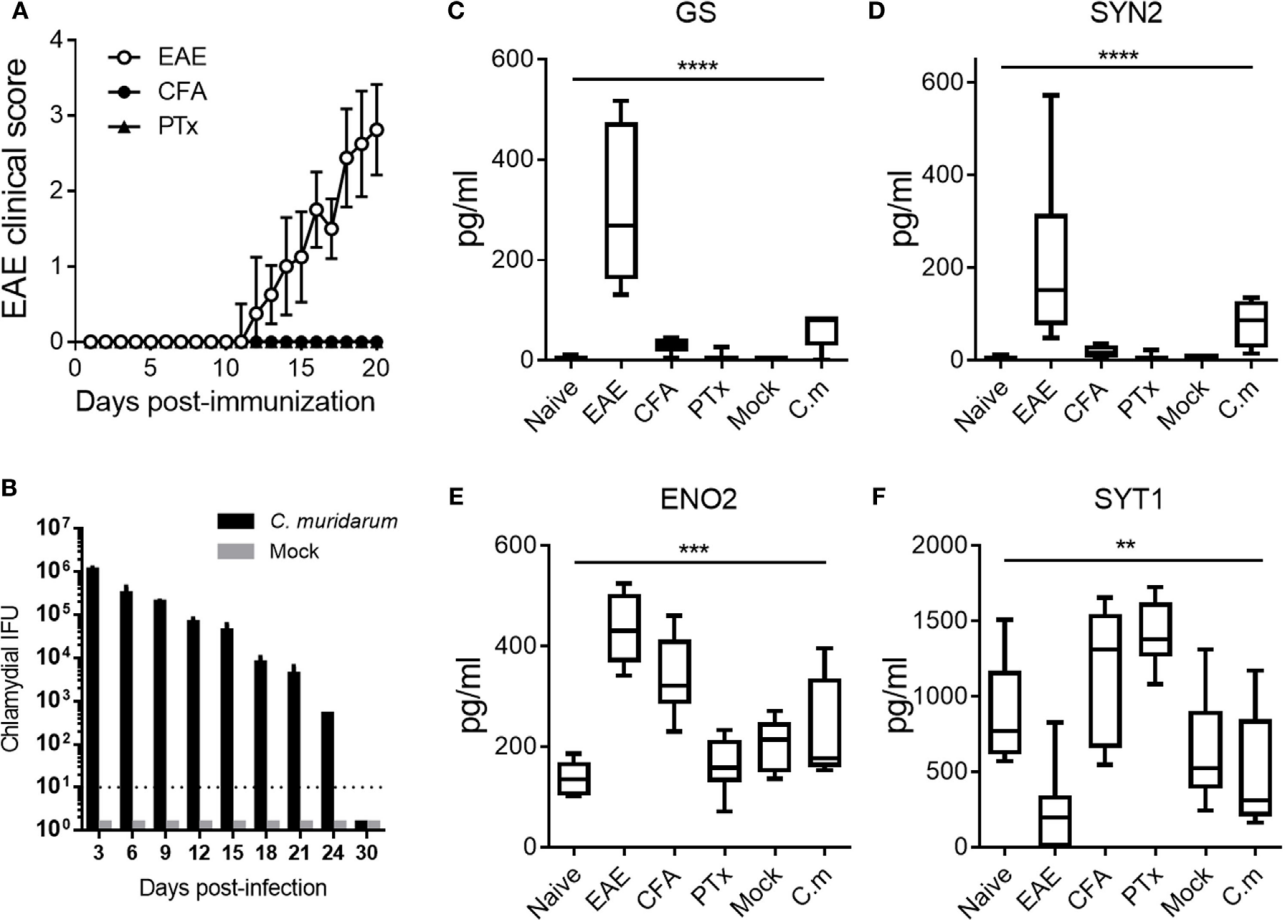
Effect of adjuvants and infection on serum neuroinflammatory disease-induced central nervous system protein expression. **(A)** Experimental autoimmune encephalomyelitis (EAE) clinical disease scores of C57BL/6 mice immunized for EAE (open circles), complete Freund’s adjuvant (CFA) immunized (closed circles), and pertussis toxin (PTx) injected controls (closed triangles). Data are shown as the mean clinical scores ± SD; *n* = 5–10 mice per group. **(B)** Inclusion-forming unit of *Chlamydia muridarum* (C.m.) measured in mice infected with C.m. (black bars) or mock infected (gray bars). Data are shown as the mean IFU ± SD; *n* = 3–5 per group per time point. **(C–F)** Serum protein abundance measured by enzyme-linked immunosorbent assay at days 5 and 7 post-challenge in naive controls, EAE mice, CFA-immunized mice, PTx-immunized mice, mock-infected mice, and C.m.-infected mice. Data are shown as boxplots with whiskers from minimum to maximum. Shown are results pooled from two to three independent experiments with *n* = 5–10 mice per group (ANOVA, *P* value: **≤0.01; ***≤0.001; ****≤0.0001).

As shown in Figure 5A, clinical disease became apparent by 11 days post-immunization in the EAE group (open circles), whereas CFA (closed circles) and PTx (closed triangles) immunized mice did not develop clinical EAE. Shown in Figure 5B is the bacterial burden at the indicated time points post-infection with *C. muridarum* (black bars) and mock (gray bars). Bacterial burdens after infection with *C. muridarum* were self-limiting and decreased over time as previously reported (17). Shown in Figures 5C–F are the serum levels of the EAE onset-leading NDICPs measured in these animals.

The results show that injection with CFA and PTx had negligible effects on serum levels of GS and SYN2 (Figures 5C,D). Moreover, *C. muridarum* infection had minor and statistically not significant effects on the serum abundance of GS and SYN2 (Figures 5C,D). Similarly, PTx injection had no significant effect on ENO2 expression. Furthermore, CFA injection or *C. muridarum* infection resulted in increased levels of serum ENO2, but the increase was of considerably lower magnitude as compared with that observed in EAE mice (Figure 5E). Importantly, these serum levels were significantly different in EAE mice as compared with control mice. Serum levels of SYT1 were not significantly affected by *C. muridarum* infection. In contrast, CFA immunization or PTx injection increased SYT1 serum expression, whereas SYT1 expression decreased during EAE (Figure 5F).

Taken together, the results show that the effect of adjuvants (i.e., CFA and PTx) on serum abundance of GS and SYN2 was negligible in our studies. Moreover, levels of GS and SYN2 were not affected by infection with *C. muridarum*. Similarly, the serum expression of SYT1 remained relatively unaffected by *C. muridarum* infection. In contrast, serum levels of ENO2 and SYT1 were affected by the CFA adjuvant in our model, and SYT1 serum levels were affected by PTx as well. Finally, the expression of ENO2 was affected by peripheral infection with *C. muridarum*. Nonetheless, the levels of serum NDICPs were statistically different in EAE mice compared with control animals. Thus, for translational purposes, it will be important to consider the effects of infection or other non-specific stimuli on the expression of potential biomarkers for MS.

### EAE Induced by Adoptive Transfer of Pathogenic Th17 Cells Induces Distinct EAE Onset-Leading NDICPs

Th17 cells are strongly associated with the pathogenesis of many autoimmune diseases including EAE and MS (34). Therefore, we determined the effect of EAE induced by adoptive transfer of Th17 cells on the serum levels of GS, SYN2, ENO2, and SYT1 in our model.

**Figure 6:**
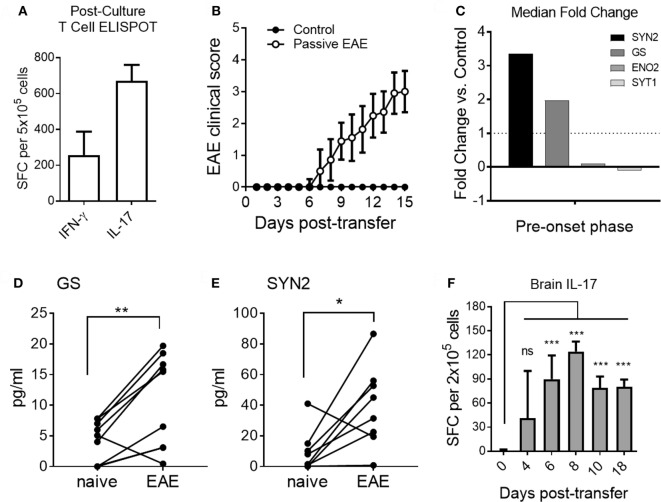
Distinct pre-onset serum neuroinflammatory disease-induced central nervous system protein wave in adoptive transfer experimental autoimmune encephalomyelitis (EAE). **(A)** Frequencies of Th1 and Th17 cells pre-adoptive transfer as measured by ELISPOT assay for IFN-γ and IL-17, respectively. Data are shown as the mean number of cytokine-producing cells ± SD. **(B)** EAE clinical disease scores of passive immunization of induction (open symbols) and control mice (closed symbols). Data are shown as the mean clinical scores ± SD **(C)** Median serum fold change for synapsin-2 (SYN2), glutamine synthetase (GS), enolase-2 (ENO2), and synaptotagmin-1 (SYT1) at days 3 and 4 post-transfer. **(D,E)** Shown is serum protein abundance measured by enzyme-linked immunosorbent assay before and after disease induction at the pre-onset phase (days 3 and 4 post-transfer) of EAE. Data are shown as longitudinal individual measurement (Student’s paired *t*-test, *P* value: *<0.05, **<0.01). **(F)** Th17 cell responses in brain of EAE mice at different time points. Shown are the frequencies of IL-17-producing T cells measured by ELISPOT assay. Data are shown as the mean number of cytokine-producing cells ± SD. Shown are results of three independent experiments with *n* = 5–12 for EAE; *n* = 3–5 for control (Student’s *t*-test, *P* value: ns, not significant; ***<0.001).

As shown in Figure 6A, MOG_35–55_-reactive donor T cells predominantly produced IL-17 after *in vitro* culture under Th17-inducing conditions prior to adoptive transfer. Upon adoptive transfer of these Th17 cells to naive recipient mice, clinical signs of EAE became apparent by day 7 post-transfer in agreement with previous studies (Figure 6B) (35). Based on our results from the active EAE model, which showed altered serum expression levels for GS, SYN2, ENO2, and SYT1 at the pre-onset phase of disease (Figures 3 and 4), we tested the serum abundance of these proteins at the pre-onset phase of passive EAE. Serum levels of GS, SYN2, ENO2, and SYT1 were determined at regular intervals before and after adoptive transfer. Of note, increased serum abundance was observed for SYN2 and GS but not for ENO2 and SYT1 at day 4 post-adoptive transfer (Figure 6C). Specifically, the serum levels for GS and SYN2 were significantly increased longitudinally at the pre-onset phase of the disease (Figures 6D,E). Furthermore, the changes in EAE onset-leading serum NDICPs coincided with the emergence of pathogenic Th17 in the CNS at this stage (Figure 6F).

Taken together, the results show that under conditions where adjuvant-mediated signals were absent, neuroinflammation caused by myelin-specific pathogenic Th17 cells promoted expression of SYN2 and GS during the pre-onset phase of EAE. Moreover, although EAE can be induced by both Th1 and Th17 cells (36), the results suggested that Th17-mediated EAE induction resulted in distinct pathophysiological mechanisms which resulted in upregulation of SYN2 and GS, but not ENO2 and SYT1.

## Discussion

In the present work, we investigated potential protein biomarkers to predict onset of clinical disease in the preclinical active EAE model by combining an unbiased M2 proteomics platform with a systems-biology “omics” data analysis strategy, consisting of statistical and bioinformatic analyses, as previously proposed (15, 37, 38). We found that in the preclinical active disease EAE model, the relative abundance of GS, SYN2, and ENO2 was increased and the relative abundance of SYT1 was decreased several days before clinical signs of EAE became apparent. Candidate protein biomarkers to forecast onset of clinical EAE were subsequently tested in studies with investigators being blinded to the clinical disease scores to provide a more clinically applicable setting and verify their potential. Furthermore, in EAE induced *via* adoptive transfer of encephalitogenic Th17 cells (passive EAE), we observed changes in serum levels of SYN2 and GS, but not ENO2 or SYT1, during the pre-onset phase of the disease. These results revealed previously unrecognized differences in NDICPs expression in active versus passively induced EAE, which may suggest key differences in pathogenic mechanism involved Th17- versus Th1/Th17-mediated neuroinflammation. Based on our results, we posit that these preclinical studies in individual subjects provide proof-of-concept for clinical measurements of statistically significant changes in serum levels of NDICPs as biomarkers for early detection of clinical signs of MS in individual patients.

Translation of potential biomarkers to human patients must take into consideration the effects of adjuvants and microbial infection in the models under study, since it is conceivable that inflammatory triggers such as peripheral infections may affect the patient’s serum levels of biomarker candidates. While we have provided some information along these lines in the EAE model with our studies, this question will have to be addressed in more extensive studies in human MS patient populations in the future. Moreover, our preclinical studies aim at MS patients where the diagnosis has been established previously clinically and by MRI; thus, it is less likely that secondary CNS disease processes, such as Parkinson disease may affect the interpretation of biomarkers for MS onset/progression. Along these lines, we observed that the serum levels of the EAE onset-leading NDICPs showed that the levels of GS and SYN2 remained relatively unaffected by adjuvants and infection, whereas ENO2 and SYT1 expression was more susceptible to modulation by these confounding factors. Last, we showed that induction of EAE *via* adoptive transfer of encephalitogenic Th17 cells resulted in a distinct expression profile of NDICPs in serum focused on SYN2 and GS but not on ENO2 and SYT1. Furthermore, the magnitude of the serum NDICP wave was greater in active EAE versus Th17 cell-induced passive EAE. Thus, it is conceivable that adjuvants such as CFA and PTx (and potentially infection) promoted enhanced serum abundance of NDICPs, for example, by activating innate immune cells *via* toll-like receptor agonists. However, it is also conceivable that the mixed Th1/Th17 cytokine profile of encephalitogenic T cells induced by immunization with neuroantigen in CFA promoted different disease-inducing mechanisms as compared with passive EAE induced by adoptive transfer of Th17 polarized cells. Along these lines, encephalitogenic T cells induced by CFA and PTx show a robust Th1 response in combination with Th17 cells (39, 40). Indeed, the ratio of Th1 to Th17 cells in EAE is linked to functional and pathogenic outcomes, such as CNS region specificity of the autoimmune response (41). For instance, Th1 cells facilitate the entry of Th17 cells into the CNS in EAE, which may initiate earlier and more vigorous neuronal damage at early stages of disease (42). Furthermore, Th1 but not Th17 cells damage astrocytes in a contact-dependent manner (39, 41). Additionally, the ratio between Th1 and Th17 cells determines the location of inflammation in the CNS and development of clinical features associated with spinal cord (high Th1 ratio) versus cerebellar and brain pathology (high Th17 ratio) (43, 44). Interestingly, GS is highly expressed in cells of the cerebral cortex and in perivascular astrocytes (45, 46), and therefore, Th1 cell-mediated damage to astrocytes may promote the release of GS into the circulation in addition to modulating Th17 cell-mediated brain pathology. Similarly, SYN2 is predominantly expressed in the gray matter (47), and thus, its expression may be altered by both pathogenic Th1 and Th17 cells and evoked by Th1 cells. In contrast, both ENO2 and SYT1 are expressed at high levels in the spinal cord and certain brain regions (48, 49). Thus, there is a reason to suspect that the differences in NDICPs observed in active versus passive EAE may not be exclusively due to adjuvants, but that the mechanisms underlying the pathogenic CNS process may play a key role in the expression profiles of NDICPs. Along these lines, our study raises important and currently unanswered questions regarding the biological processes that underlie the induction of CNS pathology in MS, and whether these may be reflected by changes in serum NDICP expression in patients that could be of diagnostic, prognostic, and therapeutic utility.

Neuronal damage accompanied by increased BBB permeability is a characteristic pathognomonic feature of MS (50, 51). Therefore, it is conceivable that inflammatory damage to neurons and/or increased vascular permeability of the BBB could result in the leakage of NDICPs into the blood circulation (33, 52). Neuronal damage and loss of BBB integrity prior to clinical disease onset were reported previously in the EAE model (33, 53). Furthermore, damage to axons, axonal transection, and neuronal death were described during EAE onset, and these pathogenic processes are therefore likely initiated prior to clinical disease symptoms (51, 54, 55). Moreover, damage to neurons at the early stages of EAE is accompanied by the degradation or loss of synapsins from neuronal cell bodies (54). Additionally, changes in neuronal plasticity and activity triggered by neuronal pathology have been reported to modulate immune responses in the CNS and neuronal proteins have been reported to modulate tissue-specific inflammation (56). Thus, it is conceivable that the reported NDICPs function in early “alarm sensing” mechanisms to initiate or modulate neuroinflammation (57). Therefore, the apparent alterations in serum NDICP abundance prior to disease onset may reflect disease activity corresponding to early neuronal damage and BBB breakdown. Consistent with this view, we reported previously on increased serum levels for other molecules during early phases of EAE, including proteins of the 14-3-3 and synapsin families (14, 15). Further support for this notion stems from the increased serum level of ENO2 (also in humans) during neurological diseases, which are accompanied by neuronal cell death and BBB damage (52, 58). Notably, 14-3-3 and ENO2 have been previously proposed as biomarkers for neuronal cell death and BBB damage in MS (59). Moreover, altered serum (and CSF) levels of ENO2 were reported in patients with clinically isolated syndrome (CIS) (60). These observations are consistent with our observations and suggest that altered serum levels of ENO2 (and other NDICPs) may be detectable at early stages of disease and allow clinical predictions.

It is an interesting conundrum that the serum levels of NDICPs in our study returned to baseline levels during the onset-lagging phase, while levels of pathogenic Th17 cells and subsequent neuronal damage continued to increase. This observation may be explained by the complex pathobiology underlying the changes in serum NDICPs, which may be driven by additional mechanisms besides neuronal damage and increased BBB permeability. For example, concurrent repair and/or compensatory mechanisms may affect the generation and leakage of NDICPs across the BBB (61). Along these lines, we noted a distinct crest of expression for SYN2 and GS (days 5 and 7, respectively), which differed from the crest expression of ENO2 and SYT1 (both days 5 and 7). Moreover, adoptive transfer of Th17 cells exclusively elicited serum expression of SYN2 and GS. These observations support the view that more than one mechanism can drive MS pathology and could potentially explain why serum levels of ENO2 in MS patients did not correlate with disease severity and progression and were decreased in patients with CIS (60). Conceivably, the EAE onset-leading serum NDICPs are the result and/or reflect early CNS repair/protective mechanisms or pathologic events during neuroinflammation, which preceded overt clinical disability. In support of this view, serum abundance of GS was increased during the pre-onset phase of EAE and declined following disease onset. GS is an enzyme which is expressed by astrocytes and is involved in glutamate uptake and metabolism. Glutamate is an excitatory neurotransmitter in the CNS, normally released under steady-state conditions. Damaged/stressed neurons in MS (and EAE) release excessive levels of glutamate, which promote neuronal injury *via* glutamate excitotoxicity, and this is a pathognomonic feature of the disease (62–64). Moreover, glutamate is also released by cells of the immune system in the CNS, including neutrophils and microglia/macrophages, which may be a contributing factor to neuronal damage (65). Thus, astrocytes may increase GS levels to balance and clear excess synaptic glutamate and thereby attempt to protect neurons from injury in EAE/MS (64, 66). Similarly, the decrease in SYT1 levels may suggest a possible role in CNS inflammation. SYT1 is involved in maintaining the pool of preformed synaptic vesicles ready for release (67). Thus, decreased levels of SYT1 may indicate the reduction of new synaptic vesicle formation in neurons, possibly because of neuronal loss and/or a decreased need for neurotransmitter release due to neuronal insult and inflammatory milieu. The increased levels in SYT1 during the clinical phase (and prior to remission) may therefore indicate the restoration of neuronal plasticity (68).

Cytokines may also play a role in altering serum levels of NDICPs through various mechanisms. For instance, IFN-γ, a Th1 cytokine, has both disease-promoting and protective functions in EAE, while the Th17 cytokines IL-17 and GM-CSF are usually viewed as disease promoting (34). Along these lines, we have recently reported that IFN-γ signaling potentiates the uptake and removal of myelin debris from the CNS by antigen presenting cells (APC) in EAE (69). It is therefore conceivable that APC export neuronal debris from the CNS to the circulation in an IFN-γ (Th1)-dependent manner and contribute to increased abundance of serum NDICPs. This may explain different abundance of serum NDICPs expression in induction of EAE by adoptive transfer of Th17-skewed T cells versus active EAE.

Taken together, the changes in serum abundance of onset-leading NDICPs may reflect differences in the etiology and pathogenic mechanisms of neuroinflammatory diseases and indicate variability in neuronal and glial function and plasticity in response to the autoimmune insult. Investigating the pattern of NDICP expression may therefore provide novel insights into CNS neuroinflammatory mechanisms and provide potential treatment targets. We have discovered high-confidence serum NDICP expression waves and protein biomarker candidates with an altered expression prior to the development of clinical signs of EAE in mice. Since GS, SYN2, ENO2, and SYT1 have human homologs, these molecules might be useful as potential biomarkers to predict the onset of relapses in RRMS patients. Moreover, these markers might be useful to monitor the effect of therapeutic drugs on CNS neuroinflammation. Conceivably, these markers might reveal subclinical disease activity, even in the absence of overt clinical signs, where tests for these markers might be an attractive and cost-efficient alternative for disease monitoring as practiced in the current standard of care. Our results suggest that it might be necessary to monitor more than one serum NDICP over the course of disease due to differences in the etiology and pathogenic mechanisms of individual patients. Nevertheless, applying high-confidence serum NDICP expression waves and protein biomarkers in a clinical setting might promote personalized drug treatment and improve efficacy.

## Ethics Statement

All animals were maintained in specific pathogen-free conditions in the American Association for Laboratory Animal Science (AALAS)-accredited facility at the University of Texas at San Antonio. All experiments were approved by the Institutional Animal Care and Use Committee (IACUC) of the University of Texas at San Antonio and performed in accordance with the relevant guidelines and regulations. Mice were fed and watered *ad libitum*.

## Author Contributions

Conceptualization: IR, WH, and TF. Methodology: IR, WH, and TF. Supervision: TF. IR, JW, FR, CH, and RG performed the experiments and collected the data. IR, JW, YW, WH, and TF designed the experiments and analyzed the data. YW, BA, WH, and TF provided reagents, a substantial intellectual contribution, and critical review of the manuscript. IR, RG, YW, BA, WH, and TF reviewed and/or revised the manuscript. IR and TF wrote the manuscript.

## Conflict of Interest Statement

The authors declare that the research was conducted in the absence of any commercial or financial relationships that could be construed as a potential conflict of interest.

## Abbreviations

NDICP, neuroinflammatory disease-induced CNS protein; RRMS, relapsing–remitting multiple sclerosis; EAE, experimental autoimmune encephalomyelitis; M2, microwave and magnetic; MOG, myelin oligodendrocyte glycoprotein; CFA, complete Freund’s adjuvant; PTx, pertussis toxin.
