# Supplementary material for: Serum Neuroinflammatory Disease-Induced Central Nervous System Proteins Predict Clinical Onset of Experimental Autoimmune Encephalomyelitis
Source: Front Immunol. 2017 Jul 17;8:812. doi: 10.3389/fimmu.2017.00812 (PMC5512177; doi:10.3389/fimmu.2017.00812)
Supplement: Supplementary file 1 [file Data_Sheet_1.docx]

Supplementary Material

**Serum Neuroinflammatory Disease-Induced CNS Proteins Predict Clinical Onset of EAE**

**Itay Raphael^1,2^, Johanna Webb^1^, Francisco Gomez-Rivera^1^, Carol A. Chase Huizar^1^, Rishein Gupta^1^, Bernard P. Arulanandam^1^, Yufeng Wang^1^, William E. Haskins^1,3^ and Thomas G. Forsthuber^1,3^**

^1^ Department of Biology, University of Texas at San Antonio, One UTSA Circle, San Antonio, TX 78249, USA.

^2^ Division of Rheumatology and Clinical Immunology, Department of Medicine, University of Pittsburgh, 3500 Terrace St, Pittsburgh, PA 15261, USA.

^3^ These authors contributed equally to this work.

**Correspondence:**

Thomas G. Forsthuber, University of Texas at San Antonio, Department Biology, One UTSA Circle, San Antonio, TX 78249, USA. Phone: 210-4585760; E-mail: [thomas.forsthuber@utsa.edu](mailto:thomas.forsthuber@utsa.edu)

**Supplementary Figures**

Supplemental Figure 1

**
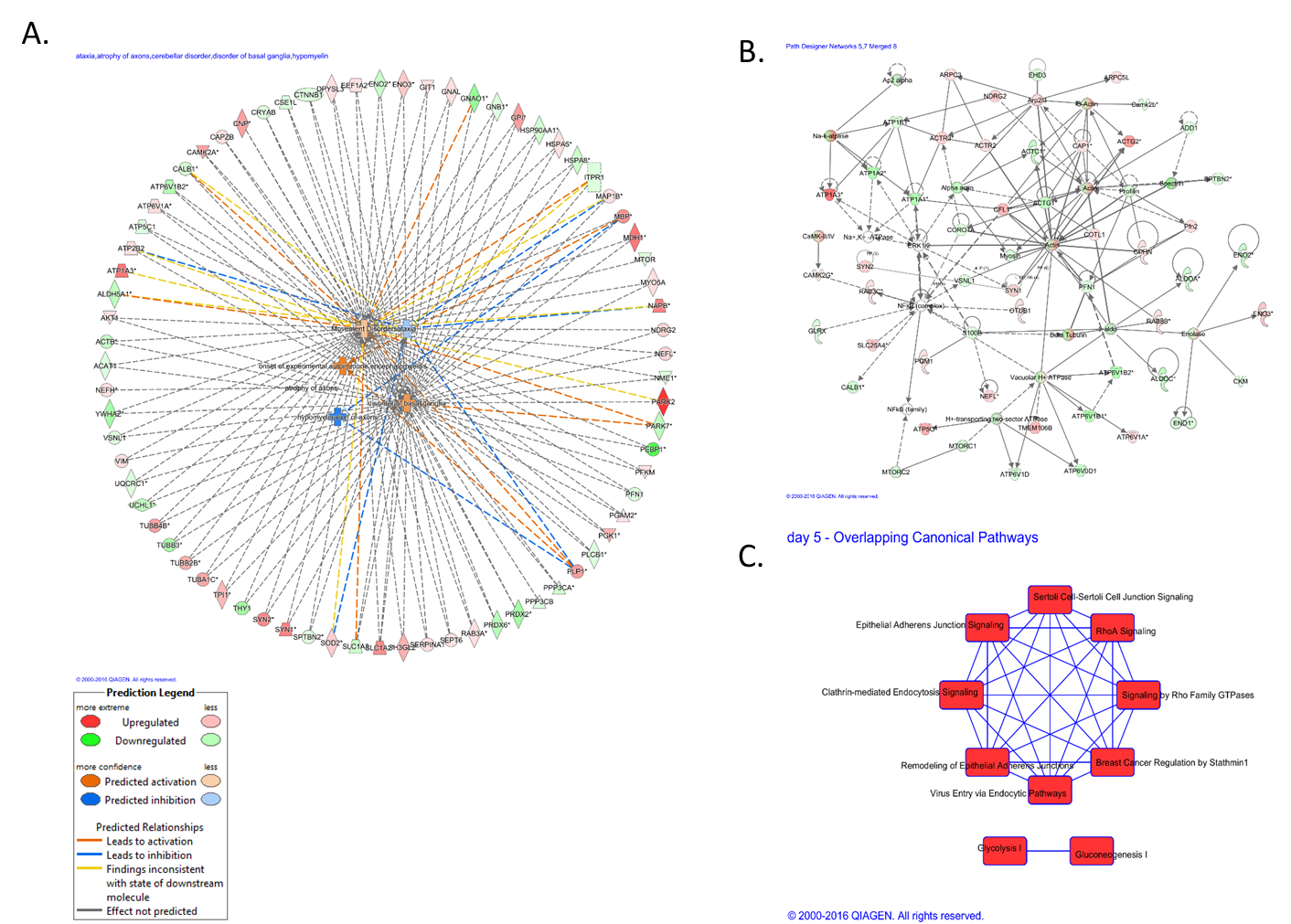
**

**Supplemental Figure 1: Identification of function-based protein biomarker candidates.**

Function, network and pathway analysis were performed using QIAGEN IPA database as described in Methods. (**A**) The top ‘disease and function’ category (neurological disease; 131 molecules; *P* value: 6.50E-03 – 7.40E-22) for day 7 post-immunization was analyzed for the most relevant functional annotations and then displayed as network. Shown is radial view for the molecules enriched in the network of ‘disorder of basal ganglia’ (*P=*7.40E-22), movement disorder (*P=*3.81E-20), ataxia (*P=*2.38E-04), atrophy of axons (*P=*1.36E-03), cerebellar disorder (*P=*2.94E-03), onset of EAE (4.40E-03), and hypomyelination of axons (*P=*5.50E-03). (**B**) Network analysis for day 7. Shown is a merged organic view of proteins enriched in two relevant networks: Network 5: Carbohydrates Metabolism, Hereditary Disorder, Neurological Disease. Network 7: Free radical Scavenging, Molecular Transport, Inflammatory Disease. (**C**) Top 10 most enriched overlapping canonical pathways at and day 5 post-immunization. Shown is a circular view of the pathways with *P* values.

Supplemental Figure 2


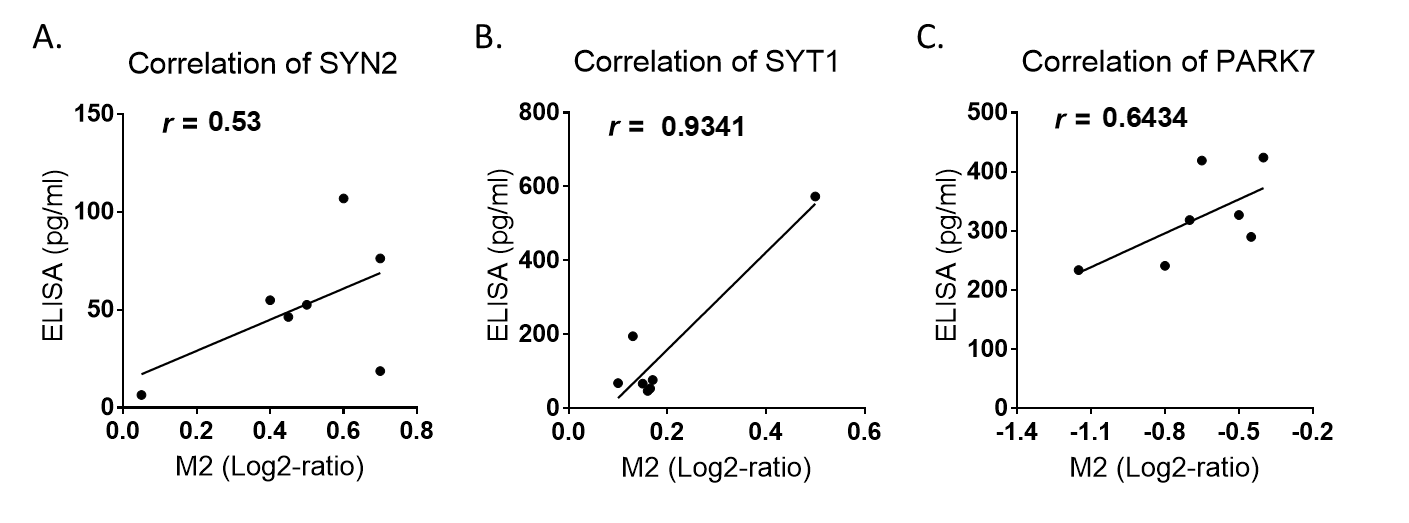


**Supplemental Figure 2: Protein expression trajectories correlation.** Linear regression correlation line for protein expression trajectories for (**A**) SYN2, (**B**) SYT1, and (**C**) PARK7.

Supplemental Figure 3


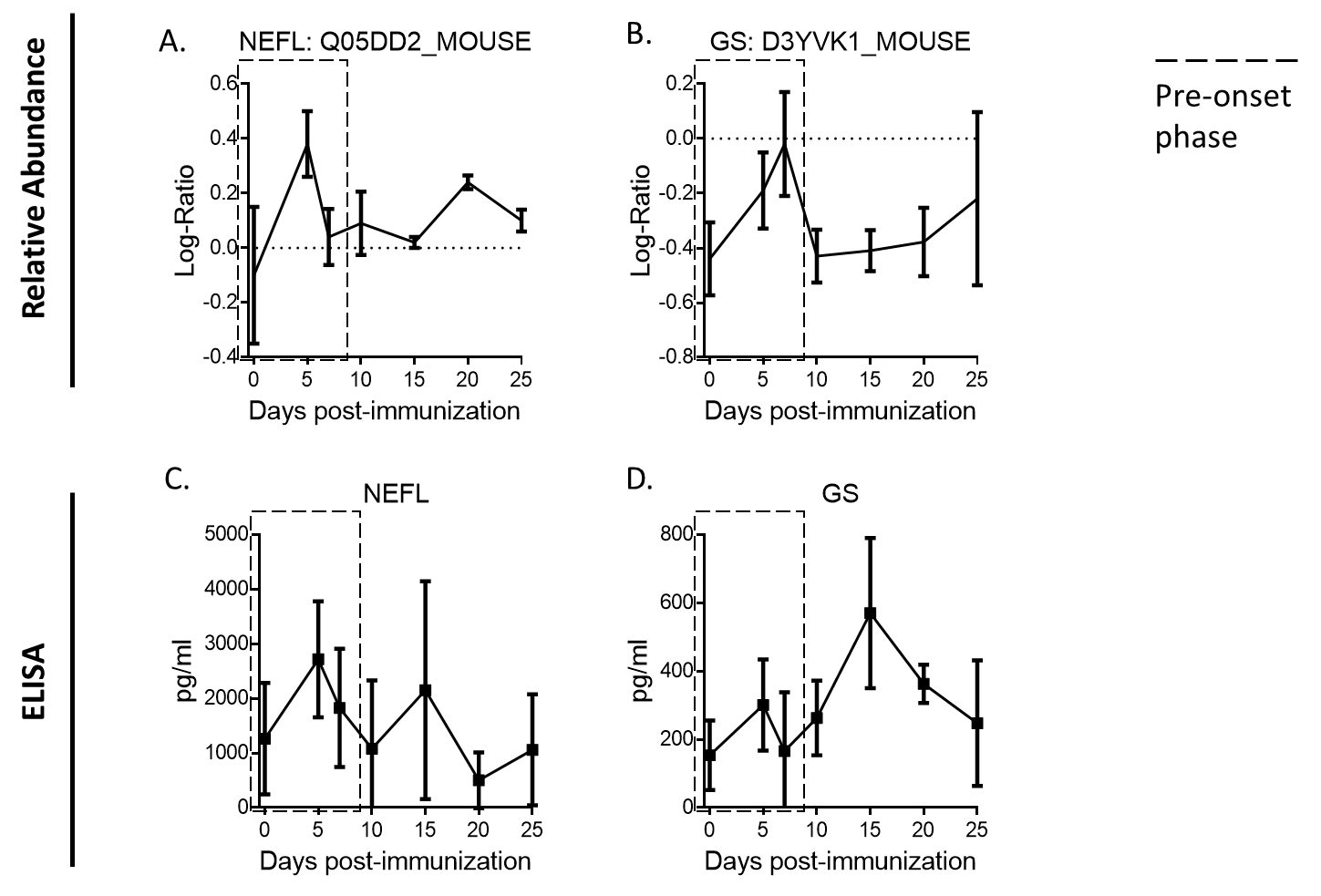


**Supplemental Figure 3: M2 proteomics protein expression trajectories correlate to protein concentration values in the CNS during EAE.** (**A, B**) Protein relative abundance in CNS tissue measured by MS/MS during EAE for (**A**) NEFL; Q05DD2_MOUSE and (**B**) GS; Q3YVK1_MOUSE. Data are shown as mean ± SD. Shown are results of one independent experiment; *n* = 20 mice per time point. (**C, D**) Protein abundance in CNS tissue measured by ELISA during EAE for (**C**) NEFL and (**D**) GS. Data are shown as mean ± SD. Shown are results of 2 independent experiments; *n* = 5 – 15 per time-point.

Supplemental Figure 4

**
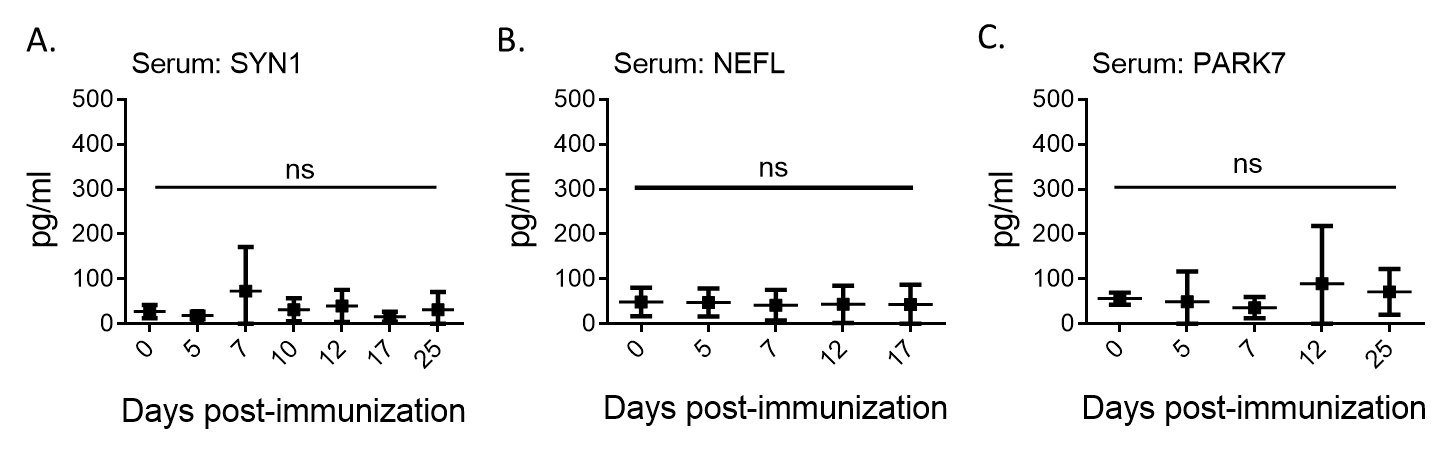
**

**Supplemental Figure 4: Serum abundance of SYN1, NEFL, and PARK7 is not significantly altered over the course of EAE.** Serum protein abundance measured by ELISA in EAE for (**A**) SYN1, (**B**) NEFL, and (**C**) PARK7. Data are shown as mean ± SD. Shown are results of 2 independent experiments; *n* = 5 – 15 per time-point. ns= not significant.

Supplemental Figure 5


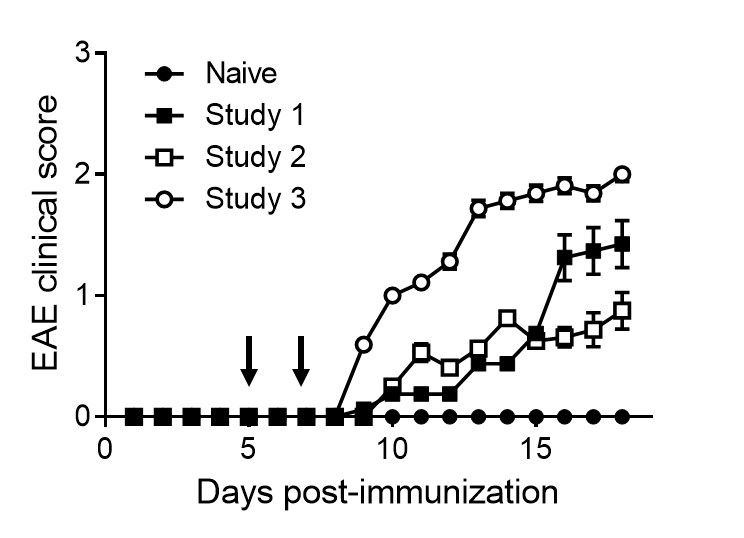


**Supplemental Figure 5: Validation of disease onset-leading CSPs in EAE mice.** Shown are the clinical disease scores for C57BL/6 mice immunized for EAE (open circles, *n* = 20 mice; open squares, *n* = 10 mice; and closed squares, *n* = 8 mice), and naïve controls (closed circles, *n* = 4 mice per group). Shown are the mean clinical scores ± SEM; Arrows indicate serum collection time points (d5 and d7 post-immunization).
